# Fetuin-A Inhibits Placental Cell Growth and Ciliogenesis in Gestational Diabetes Mellitus

**DOI:** 10.3390/ijms20205207

**Published:** 2019-10-21

**Authors:** Chia-Yih Wang, Mei-Tsz Su, Hui-ling Cheng, Pao-Lin Kuo, Pei-Yin Tsai

**Affiliations:** 1 Department of Cell Biology and Anatomy, College of Medicine, National Cheng Kung University, Tainan 701, Taiwan; b89609046@gmail.com (C.-Y.W.); tomato4329@gmail.com (H.-l.C.); 2 Institute of Basic Medical Sciences, College of Medicine, National Cheng Kung University, Tainan 701, Taiwan; 3 Department of Obstetrics and Gynecology, National Cheng Kung University Hospital, College of Medicine, National Cheng Kung University, Tainan 704, Taiwan; sumeitsz@mail.ncku.edu.tw (M.-T.S.); paolinkuo@gmail.com (P.-L.K.)

**Keywords:** fetuin-A, GDM, cell growth, centrosome, primary cilium, autophagy

## Abstract

Gestational diabetes mellitus (GDM) is a type of unbalanced glucose tolerance that occurs during pregnancy, which affects approximately 10% of pregnancies worldwide. Fetuin-A is associated with insulin resistance, and the concentration of circulating fetuin-A increases in women with GDM, however, the role of fetuin-A in the placenta remains unclear. In this study, we enrolled placental samples from twenty pregnant women with GDM and twenty non-GDM pregnant women and found that the abundance of fetuin-A was upregulated in terms of mRNA and protein levels. Fetuin-A inhibited placental cell growth by inducing apoptosis and inhibiting S phase entry. Irregular alignment of mitotic chromosomes and aberrant mitotic spindle poles were observed. In addition, centrosome amplification was induced by fetuin-A treatment, and these amplified centrosomes nucleated microtubules with disorganized microtubule arrays in placental cells. Furthermore, fetuin-A inhibited autophagy, and thus blocked the growth of the primary cilium, a cellular antenna that regulates placenta development and differentiation. Thus, our study uncovered the novel function of fetuin-A in regulating placental cell growth and ciliogenesis.

## 1. Introduction

Gestational diabetes mellitus (GDM) is a type of unbalanced glucose tolerance that occurs during pregnancy, which affects approximately 10% of pregnancies worldwide [1]. The clinical and public health relevance of gestational diabetes mellitus has been widely debated due to its increasing incidence and resulting negative economic impact, and the potential for severe GDM-related pregnancy complications [2]. In addition, the American Diabetes Association (ADA) “Management of Diabetes in Pregnancy: Standards of Medical Care in Diabetes 2019” recommends diabetes care from preconception to postpartum [3]. Several risk factors, including obesity, history of previous GDM diagnosis, advanced maternal age, and gestational hypertension, have been implicated in the pathogenesis of GDM [4,5]. In general, specific risks of uncontrolled diabetes in pregnancy include spontaneous abortion, fetal anomalies, preeclampsia, fetal demise, macrosomia, neonatal hypoglycemia, and neonatal hyperbilirubinemia, among others. In addition, diabetes in pregnancy may increase the risk of obesity and type 2 diabetes in the offspring later in life [6,7]. Thus, it is important to understand the pathogenesis of GDM.

Fetuin-A belongs to the cystatin protease inhibitor superfamily [8]. It is the major human secretory protein derived from the liver and adipose tissue and performs several pathophysiological functions related to insulin sensitivity [9], glucose tolerance [10], and even soft tissue calcification [11]. In the liver and skeletal muscles, fetuin-A, per se, is an endogenous inhibitor of the insulin receptor [12] and is crucial for lipid-induced insulin resistance [13]. By binding to the β-subunit of the insulin receptor, fetuin-A inhibits the activity of the insulin receptor, followed by the blocking of insulin-stimulated GLUT4 translocation and Akt activation [14]. In addition, fetuin-A is also involved in inflammatory signaling [13]. Fetuin-A acts as an endogenous ligand for the innate immune Toll-like receptor (TLR)-4, thus promoting lipid-induced insulin resistance. Moreover, fetuin-A is associated with several metabolic disorders. High serum fetuin-A concentrations are observed in patients with several metabolic syndromes, including insulin resistance, fatty liver, and diabetes [10,15]. A recent study also showed that the circulating fetuin-A concentration increases in GDM women [16], however, the underlying molecular mechanism is still unclear.

The centrosome is the major microtubule organization center that orchestrates microtubule networks for proper cell migration and division [17]. It comprises mother and daughter centrioles and the surrounding pericentriolar materials (PCM). The duplication of the centrosome coordinates with DNA replication. During the S phase, each centriole functions as a platform for a new procentriole to grow. The duplicated centrosomes start to separate to the opposite site of the nucleus, followed by the establishment of mitotic spindle poles for proper chromosome segregation in the M phase. Thus, precise control of centrosome homeostasis is important to maintain cell growth and genomic instability [18,19].

The centrosome also contributes to the growth of the primary cilium [20]. The primary cilium is the cellular protrusion that receives chemical or mechanical signals for proper development and differentiation [21]. The primary cilium is composed of the central microtubule-built axoneme and the overlying ciliary membrane [22]. On the axoneme, intraflagellar transporters regulate cilia dynamics and functions via anterograde and retrograde transportations. Recent studies have also demonstrated that the primary cilia play important roles in placentation during early pregnancy [23].

In this study, we enrolled placental samples from twenty pregnant women with GDM and twenty non-GDM pregnant women and found that the abundance of fetuin-A was upregulated in terms of mRNA and protein levels. The upregulated fetuin-A impeded cell cycle progression and induced apoptosis. In addition, centrosome amplification with disorganized microtubule arrays was observed in fetuin-A-treated placental cells. Furthermore, fetuin-A inhibited autophagy, therefore, blocking the growth of the primary cilium. Thus, our study uncovered the effect of fetuin-A on the regulation of placental cell growth and ciliogenesis.

## 2. Results

### 2.1. The Expression of Fetuin-A Is Upregulated in the Placentas of Gestational Diabetes Mellitus (GDM) Patients

**Figure 1:**
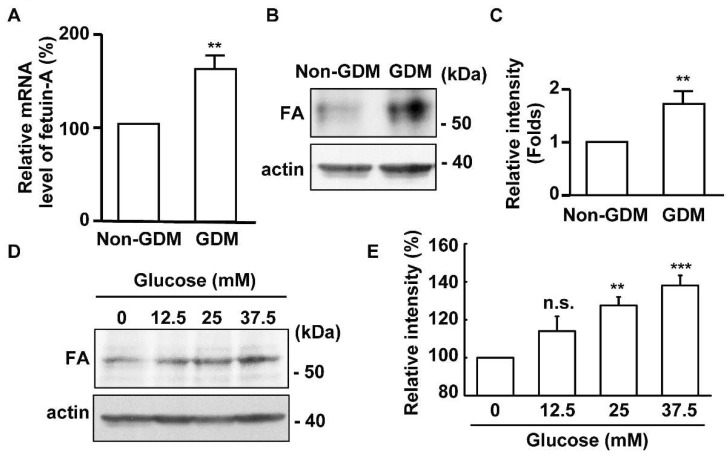
Fetuin-A is upregulated in the placentas of gestational diabetes mellitus (GDM) patients. (**A**–**C**) Fetuin-A is upregulated in the placentas of GDM patients: (**A**) quantification results of the fetuin-A mRNA level in the placentas of non-GDM and GDM women, (**B**) whole placenta extracts of non-GDM and GDM women were analyzed by immunoblot with antibodies against fetuin-A and actin, and (**C**) quantification results in (**B**). (**D**,**E**) Glucose induces the expression of fetuin-A in HTR8 cells: (**D**) whole cell extracts of fetuin-A-treated HTR8 cells were analyzed by immunoblot with antibodies against fetuin-A and actin and (**E**) quantitation of the relative intensity of fetuin-A in (**E**). n.s., no significance; ** *p* < 0.01 and *** *p* < 0.001.

Pregnant women who suffer from gestational diabetes mellitus (GDM) show higher levels of fetuin-A in their circulation [24], however, little is known about the effect of fetuin-A in the placenta. Therefore, we enrolled placental samples from twenty pregnant women with GDM and twenty non-GDM pregnant women. In the first trimester, the mean value of the body mass index (BMI) in the control group was 23.40 and that in the GDM group was 27.06. In the third trimester, the mean BMI value in the control group was 26.80 and that in the GDM group was 30.38 (Table 1). The treatment strategy for women with GDM follows the guidelines of the ADA “Management of Diabetes in Pregnancy: Standards of Medical Care in Diabetes 2019”, including lifestyle management, medical nutrition therapy, and pharmacological therapy [3]. The mRNA level of fetuin-A in the placenta, which was measured by real-time PCR, was increased in patients with GDM (Figure 1A, *p* = 0.008). To further confirm this, the protein level of fetuin-A was also analyzed by immunoblotting assay. The abundance of placental fetuin-A was higher in GDM patients than in non-GDM subjects (Figure 1B,C, *p* = 0.008). Thus, the expression of fetuin-A is upregulated in the placentas of patients with GDM. Next, we tested whether the upregulation of fetuin-A in the placenta was induced by glucose. The immortalized placental HTR8 cells were cultured with different concentrations of glucose for 72 h, and the expression of fetuin-A was examined. The abundance of fetuin-A was increased in a dose-dependent manner (Figure 1D,E, ** *p* = 0.007 and *** *p* = 0.0002). Thus, the expression of fetuin-A is induced by high glucose treatment in HTR8 cells.

### 2.2. Fetuin-A Inhibits Placental Cell Growth

**Figure 2:**
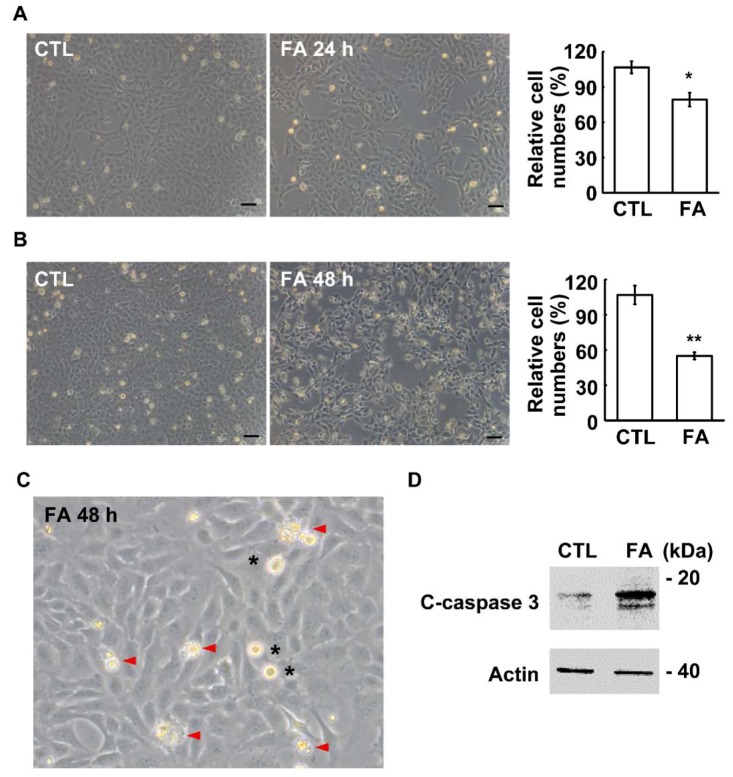
Fetuin-A inhibits HTR8 cell growth. (**A**,**B**) Fetuin-A inhibits HTR8 cell growth in a time-dependent manner. The cell numbers are shown as bright-field images (left panel) and quantification results (right panel) following treatment with 600 µg/mL of fetuin-A in HTR8 cells for 24 h (**A**) and 48 h (**B**). CTL: control and FA: fetuin-A. These results are the mean ± SD from three independent experiments. Scale bar 100 µM. (**C**,**D**) Fetuin-A induces apoptosis. (**C**) The apoptotic bodies (arrowhead in red) are observed upon treatment with 600 µg/mL of fetuin-A for 48 h in HTR8 cells. The mitotic cells are indicated by asterisks. The magnification is 400×. (**D**) Whole cell extracts of fetuin-A-treated HTR8 cell line were analyzed by immunoblot with antibodies against cleaved-caspase-3 (C-caspase-3) and actin. * *p* < 0.05 and ** *p* < 0.01.

The effect of fetuin-A on placental cell growth was examined. A previous study showed that treatment with 600 µg/mL of fetuin-A for 48 h inhibited primary extravillous trophoblast cell growth [25]. Therefore, we treated HTR8 cells with 600 µg/mL of fetuin-A for 24 or 48 h, and the cell numbers were counted. At 24 h after fetuin-A treatment, the cell numbers were significantly reduced, and treatment with fetuin-A for 48 h inhibited placental cell growth to the half maximal inhibitory concentration (IC50) (Figure 2A,B, Figure 2A: *p* = 0.04 and Figure 2B: *p* = 0.0009). Thus, the following experiments were performed by treating cells with 600 µg/mL of fetuin-A for 48 h. When checking the morphology of fetuin-A-treated cells, several apoptotic bodies were observed, suggesting that fetuin-A treatment might induce apoptosis. To further confirm this, the marker of apoptosis, cleaved-caspase-3, was checked. Upon fetuin-A treatment, the level of cleaved-caspase-3 increased significantly (Figure 2C,D). Thus, fetuin-A induces apoptosis in placental cells.

**Figure 3:**
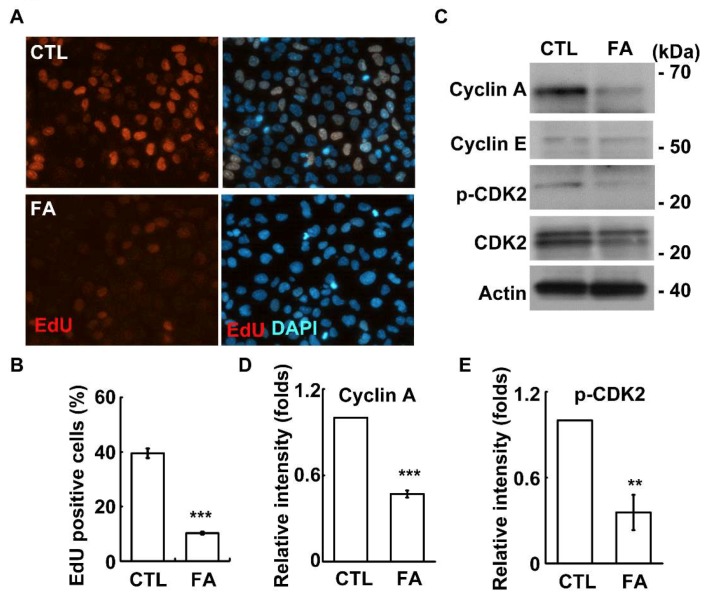
Fetuin-A inhibits S phase entry. (**A**,**B**) EdU incorporation was reduced in fetuin-A-treated HTR8 cells: (**A**) immunostaining of EdU (red) and DAPI (blue) in scramble control (CTL) or fetuin-A (FA) treated HTR8 cells and (**B**) quantification results of (**A**). The magnification is 200×. These results are the mean ± SD from three independent experiments and more than 1000 cells were counted in each individual group. (**C**–**E**) Fetuin-A inhibited cyclin A expression and CDK2 activation: (**C**) whole cell extracts of fetuin-A-treated HTR8 cells were analyzed by immunoblot with antibodies against cyclin A, cyclin E, CDK2, phosphorylated CDK2 at Thr160 (p-CDK2), and actin. (**D**,**E**) Quantitation of the relative intensity of cyclin A (**D**) and p-CDK2 in (**E**). ** *p* < 0.01 and *** *p* < 0.001.

**Figure 4:**
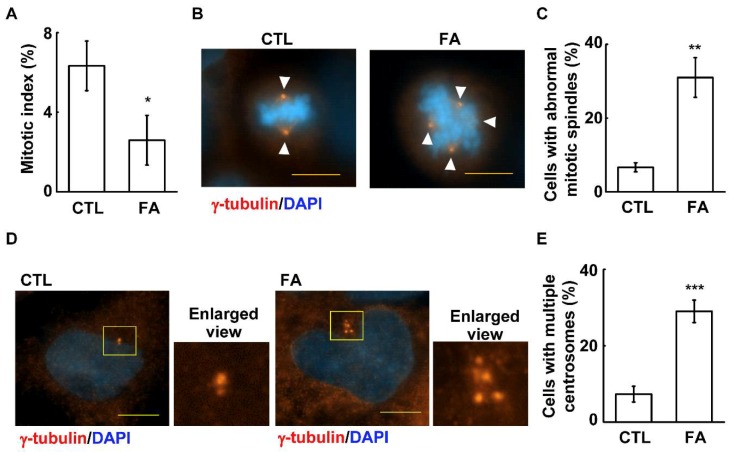
Fetuin-A induces centrosome amplification. (**A**) Fetuin-A inhibited cells entering the M phase. Quantification results of the mitotic index in the absence (CTL) or presence of fetuin-A (FA). These results are the mean ± SD from three independent experiments; more than 1000 cells were counted in each individual group. (**B**,**C**) Aberrant mitotic spindle poles were induced by fetuin-A treatment: (**B**) immunofluorescence staining showed increased mitotic spindle poles (γ-tubulin staining, as shown by the arrowhead) upon fetuin-A treatment and (**C**) quantification results of (**B**). (**D**,**E**) Fetuin-A induced centrosome amplification: (**D**) immunofluorescence staining showed increased γ-tubulin numbers upon fetuin-A treatment and (**E**) quantification results of (**D**). ** *p* < 0.01 and *** *p* < 0.001, results are the mean ± SD from three independent experiments, more than 100 cells were counted in each individual group. Scale bar 5 µM.

To further study how fetuin-A affects cell growth, the ability of cells to enter into the S phase was examined by the EdU incorporation assay. Fetuin-A treatment reduced the population of cells with EdU positive signals (Figure 3A,B, *p* = 6 × 10^−8^). Then, the S phase related cyclins, including cyclin E and cyclin A, and the activation of CDK2 were examined. The abundance of cyclin A, but not cyclin E, and the level of phosphorylated CDK2 were reduced upon fetuin-A treatment (Figure 3C–E; Figure 3D, *p* = 3 × 10^−6^, and Figure 3E, *p* = 0.002). These data suggest that fetuin-A inhibits cyclin A-CDK2 activation, and thus leads to reduced S phase entry. Next, the ability of cells to enter the M phase was examined. Upon fetuin-A treatment, the mitotic index was reduced, indicating that the ability of cells to enter the M phase was reduced (Figure 4A, *p* = 0.02). In conclusion, fetuin-A inhibits placental cell growth.

### 2.3. Fetuin-A Induces Centrosome Amplification

The mitotic apparatus of the fetuin-A-treated cells was further examined. Normally, the mitotic spindle poles (γ-tubulin signals) orchestrate the mitotic spindle to align the duplicated chromosomes in the middle of the cells (Figure 4B, left panel), however, upon fetuin-A treatment, aberrant multiple mitotic spindle poles (cells with more than two γ-tubulin spots at M phase) were observed, accompanied by chromosome misalignment (Figure 4B, right panel and Figure 4C, *p* = 0.003). Thus, fetuin-A reduces M phase entry and disorganizes the mitotic apparatus in placental cells.

As centrosomes form the mitotic spindle poles for proper chromosome segregation, abnormal centrosomes might lead to the development of aberrant mitotic spindles. Thus, the centrosome numbers were counted by the staining of the marker of pericentriolar material, γ-tubulin, in fetuin-A-treated cells. When examining the centrosomal numbers, only one (before duplication) or two (after duplication) centrosomes were observed in control cells, however, the treatment of fetuin-A led to centrosome amplification, as shown by the presence of more than two γ-tubulin spots (Figure 4D,E, *p* = 0.001). Thus, fetuin-A induces centrosome amplification.

### 2.4. Fetuin-A Leads to Disorganized Microtubule Nucleation

**Figure 5:**
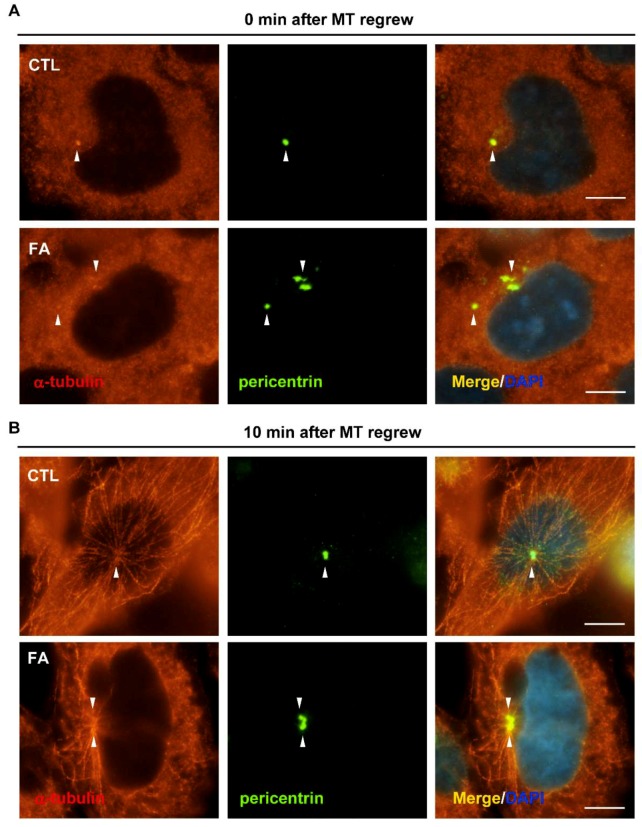
Fetuin-A treatment leads to disorganized microtubule arrays. Microtubules were depolymerized by nocodazole treatment (**A**), and, then, cells were cultured in the drug-free medium for 10 min (**B**), in the absence (CTL) or presence of fetuin-A (FA). Centrosomes and microtubules (MT) were immunostained with antibodies against pericentrin (centrosome marker) and γ-tubulin (microtubule marker). DNA was stained with DAPI. Scale bar 5 µM.

Microtubule nucleation activity is mainly regulated by the centrosome, and fetuin-A leads to aberrant centrosome homeostasis. Thus, the microtubule nucleation ability was examined by the microtubule regrowth assay. Microtubules were disrupted by 1 h of nocodazole treatment. Then, the cells were washed by PBS and grown in the fresh medium for 10 min to allow the microtubule to regrow. Nocodazole treatment efficiently disrupted microtubule arrays in both the control and fetuin-A-treated cells (Figure 5A). After microtubule regrowth for 10 min, the microtubule concentrated around the centrosome and the array started to emanate from the centrosome to the periphery of the cells (Figure 5B, upper panel), however, in fetuin-A-treated cells, the emanated microtubule density was less than that of in the control cells, and these microtubule arrays did not extend to the cell periphery (Figure 5B, lower panel). Thus, fetuin-A treatment leads to the disorganization of microtubule nucleation.

### 2.5. Fetuin-A Inhibits Primary Cilium Formation in Placental Cells

**Figure 6:**
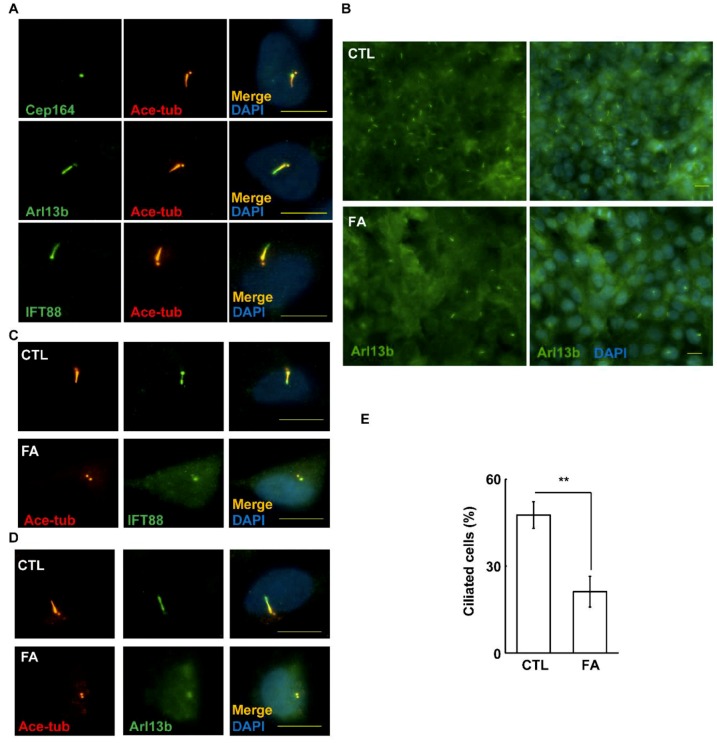
Fetuin-A inhibits ciliogenesis. (**A**–**D**) Primary cilia were examined in the absence (CTL) or presence of fetuin-A (FA) by immunostaining with antibodies against acetylated tubulin (Ace-tub), Cep164, IFT88, or Arl13b. (**E**) Quantification results of the frequency of ciliated HTR8 cells. ** *p* < 0.01, results are the mean ± SD from three independent experiments, more than 100 cells were counted in each individual group. DNA was stained with DAPI. Scale bar 10 µM.

The primary cilium is important for maintaining placenta development. Therefore, we examined whether fetuin-A affects primary cilium formation. First, we examined whether placental HTR8 cells could grow primary cilia by forcing cells to enter the G0 phase under serum deprivation. At the G0 stage, the primary cilium, as shown by acetylated tubulin staining (axoneme marker), started to grow from the mother centriole (Cep164 staining) (Figure 6A, upper panel). Then, we examined other ciliary components. The ciliary membrane marker Arl13b and the intraflagella transporter marker IFT88 colocalized with the acetylated tubulin, suggesting that these cilia were intact. Then, we examined the effects of fetuin-A on ciliogenesis. Fetuin-A treatment inhibited primary cilia formation, as shown by the reduction of all ciliary markers (Figure 6B–E, *p* = 0.005). Thus, fetuin-A inhibits primary cilium formation in placental cells.

### 2.6. Fetuin-A Inhibits Autophagic Flux

**Figure 7:**
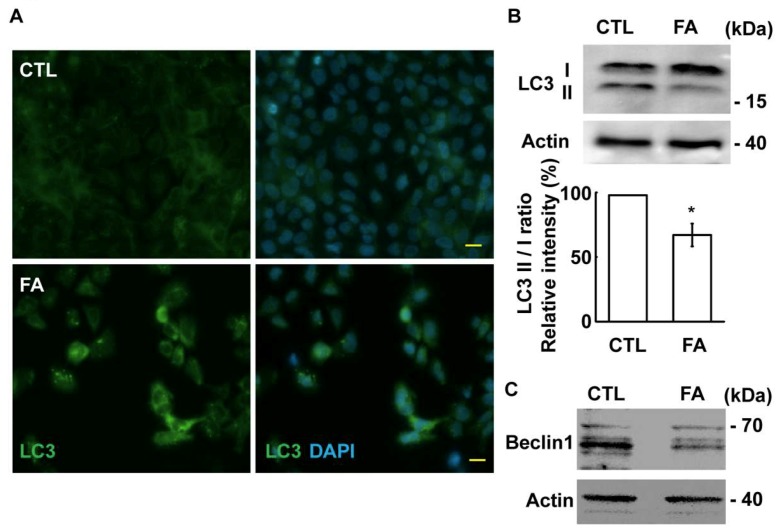
Fetuin-A inhibits autophagy. (**A**) Autophagy was examined by immunostaining with an antibody against LC3. DNA was stained with DAPI. Scale bar 10 µM. (**B**,**C**) Extracts of control (CTL) or fetuin-A (FA)-treated cells were analyzed by immunoblotting with antibodies against LC3, Beclin1, or actin. (**B**, lower panel) Quantification results of the relative intensity of the LC3 II to LC3 I ratio. * *p* < 0.05.

Autophagy promotes primary cilium formation under serum deprivation. Therefore, we examined whether fetuin-A affects autophagy. First, we examined the autophagy by immunostaining with an antibody against LC3. In the control cells, the LC3 signal was hardly detected, however, Fetuin-A treatment induced LC3 accumulation in the cytoplasm, suggesting that fetuin-A treatment affected autophagy (Figure 7A). The accumulation of LC3 signal in the cytoplasm might result from the acceleration of autophagy or defective autophagic flux, and thus the conversion of LC3 I to LC3 II was examined by immunoblotting assay. Upon fetuin-A treatment, the conversion of LC3 I to LC3 II was reduced, as shown by a lowered LC3 II to I ratio (Figure 7B, *p* = 0.02), suggesting that the autophagic flux was reduced. To further confirm this observation, the expression of Beclin1, the key enzyme in the initiation of autophagic flux, was examined. The expression of Beclin1 was reduced by fetuin-A treatment (Figure 7C). Thus, fetuin-A inhibits autophagic flux.

## 3. Discussion

In this study, we demonstrated that fetuin-A is relevant for cell growth and ciliogenesis in the placentas of GDM patients. Treatment with fetuin-A leads to abnormal centrosome amplification followed by the induction of aberrant mitotic spindle poles. In addition, fetuin-A also disorganizes the microtubule arrays. Furthermore, fetuin-A inhibits autophagy, followed by the reduction of primary cilium formation in placental cells. Taken together, fetuin-A affects the centrosome and autophagy, leading to defective placenta growth.

Circulating fetuin-A is upregulated in GDM patients [26], however, little is known about the effect of fetuin-A on the placenta. In this study, we showed that the placental fetuin-A concentration increases in GDM women. It is an important issue to clarify the source of placental fetuin-A. The elevated fetuin-A might be derived from maternal circulation. Additionally, it might also be synthesized locally at the placenta. In our study, we found that the mRNA level of fetuin-A increased in the placentas of GDM patients. In addition, a high glucose concentration induced the expression of fetuin-A in immortalized placental cells, suggesting that fetuin-A was also synthesized locally. Thus, fetuin-A might act on the placenta through paracrine or autocrine effects. Circulating maternal fetuin-A acts on the placenta via a paracrine effect. A high maternal glucose concentration also induces placental cells to produce more fetuin-A locally. Thus, both circulating and local placental fetuin-A coordinately affect placental development in GDM.

The primary cilium regulates development and differentiation and, additionally, this tiny organelle plays roles in female reproduction. Endocrine gland-derived vascular endothelial growth factor (EG-VEGF), the key endocrine factor for proper placentation, binds to its receptor on the primary cilium, triggering the downstream signaling cascade for trophoblast invasion [23,27]. The regulation of ciliogenesis is initiated by TTBK2 recruitment and CP110 removal from the mother centriole [28], and resorption of the primary cilium is triggered by HDAC6 activation [29]. Recent studies showed that the primary cilia is also regulated by the miR-200 family in the placenta [27]. In this sstudy, we showed that fetuin-A also inhibits ciliogenesis in placental cells. So far, it is still unclear how fetuin-A affects the placental cilia. The concentration of miR-200 family members is upregulated in women with GDM [30]. In addition, the concentrations of fetuin-A and miR-200 family members increase in nonalcoholic fatty liver disease patients [10,31]. Thus, we speculate that, in the placentas of GDM women, the fetuin-A might induce the expression of miR-200 family members, thus inhibiting the formation of the primary cilia, however, this hypothesis still needs to be tested.

In summary, we showed that fetuin-A inhibits placental cell growth and is required for the control of centrosome homeostasis and ciliogenesis, which are two important events for proper placentation.

## 4. Materials and Methods

### 4.1. Cell Culture

The immortalized HTR-8/SVneo (HTR8) cell line (ATCC CRL-3271), which was obtained from ATCC (Manassas, VA, USA). was derived by transfecting cells that grew out of chorionic villi explants of human first-trimester placentas with the SV40 large T antigen. HTR8 cells were grown in Roswell Park Memorial Institute (RPMI)-1640 medium supplemented with 10% fetal bovine serum. These cells were cultured in a humidified atmosphere at 5% CO_2_ at 37 °C. Mycoplasma contamination was regularly examined by immunoblotting assay and immunofluorescence staining (DNA was stained by DAPI) according to the guidelines.

### 4.2. Study Population and Sample Collection

The experimental procedure was approved by the biosafety committee of the National Cheng Kung University. The human sample analysis was approved by the Institutional Review Board (IRB) of the National Cheng Kung University Hospital (NCKUH, no. A-ER-105-021; 19 February 2016). The expression level of fetuin-A in the placentas of women with GDM (*n* = 20) and healthy controls (*n* = 20) was measured in a case-control study. The diagnosis of gestational diabetes was made at 24 to 28 weeks of gestation via a two-hour 75 g oral glucose tolerance test according to the guidelines of the IADPSG (International Association of the Diabetes and Pregnancy Study Groups) and ADA (American Diabetes Association). The positive diagnosis criteria were one or more plasma glucose values that met or exceeded the following values: fasting glucose level > 92 mg/dL, 1 h glucose level > 180 mg/dL, and 2 h glucose level > 153 mg/dL. Only singleton pregnancies were included in the study.

### 4.3. Immunofluorescence Microscopy

Cells were grown on coverslips prior to the performance of experiments. Cells were fixed with cold methanol at −20 °C for 6 min. Then, the cells were washed with PBS three times followed by blocking with 5% BSA for 1 h. After blocking, cells were incubated with antibodies overnight at 4 °C. Then, cells were washed with PBS three times and incubated with FITC-conjugated and/or Cy3-conjugated secondary antibodies (Invitrogen, Carlsbad, CA, USA) for 1 h at room temperature in the dark. After extensive washing, the cover slips were mounted in 50% glycerol (in PBS) on glass slides. Cells were observed with an AxioImager M2 fluorescence microscope (Zeiss, Switzerland).

### 4.4. Microtubule Regrowth Assay

Cells were cultured in nocodazole (10 mg/mL) containing medium for 1 h to depolymerize the microtubules. Then, the nocodazole was removed by washing with PBS three times, and then cells were incubated in fresh medium at 37 °C for 10 min. After microtubule repolymerization, cells were fixed with cold methanol at −20 °C for 6 min, followed by immunofluorescence staining.

### 4.5. EdU Incorporation Assay

Cells were cocultured with commercially available EdU for 30 min according to the manufacturer’s instructions (Invitrogen, Carlsbad, CA, USA). Then, these cells were observed with fluorescence EdU signals by an AxioImager M2 fluorescence microscope.

### 4.6. Cell Growth Assay

Cells were trypsinized and resuspended in PBS for cell number counting or they were centrifuged for further Western blot analysis after drug treatment. Centrifuged cells were further lysed with lysis buffer containing 0.5% NP-40, 300 mM NaCl, 1 mM EDTA, and the protease inhibitor cocktail (Roche, Mannheim, Germany) followed by centrifugation (15,000 rpm, 4 °C). The supernatant was collected and further analyzed by Western blot analysis.

### 4.7. Antibodies

The following antibodies were obtained commercially: anti-acetylated-tubulin (T7451), anti-γ-tubulin (T5326), and anti-α-tubulin (T9026) (Sigma, St. Louis, MO, USA); anti-LC3A/B (D3U4C) XP (#12741), anti-cyclin A2 (#4656), anti-cyclin E1 (HE12; #4129), anti-CDK2 (#2546), and anti-CDK2 phospho-Thr160 (#2561) were purchased from Cell Signaling (Beverly, MA, USA); anti-fetuin-A (ab137125), and anti-Beclin 1 antibody [EPR19662] (ab207612) were purchased from Abcam (Cambridge, UK); anti-actin (AC-15) (GTX26276) was purchased from Genetex (Irvine, CA, USA); anti-IFT88 (13967-1-AP) was purchased from Proteintech (Chicago, IL, USA); and anti-CEP164 (NBP1-81445) was purchased from Novus (Littleton, CO, USA).

### 4.8. Statistical Analysis

All data are presented as the mean ± S.D. from at least three independent experiments. In each individual experiment, more than 100 cells were counted in each experimental group. To show the statistical significance, unpaired two-tailed t-tests were used to show the differences between two groups. *p*-values of less than 0.05 were considered statistically significant.

## Figures and Tables

**Table 1:** Characteristics of study population.

	Normal Control (*n* = 20)	GDM (*n* = 20)
Maternal age (years)	32.78 ± 4.2	33.56 ± 4.93
Nulliparity (%)	41.94	48
Gestational age at delivery (weeks)	38.43 ± 1.14	38 ± 1.2
Chinese Han ethnicity (%)	100	100
BMI 1^st^ trimester (Kg/m^2^)	23.4 ± 3.48	27.06 ± 5.85 **
BMI 3^rd^ trimester (Kg/m^2^)	26.8 ± 3.39	30.38 ± 6 **
Systolic blood pressure (mmHg)	123.04 ± 12.55	133.43 ± 19.73 *
Diastolic blood pressure (mmHg)	73.83 ± 10.01	81.71 ± 11.56 *
Glucose-AC (mg/dL)	78 ± 7.01	97.74 ± 28.22
Glucose-PC 1 hour (mg/dL)	134.83 ± 6.34	195.67 ± 55.1 *
Glucose-PC 2 hours (mg/dL)	126.67 ± 26.07	193.47 ± 71.5 *
Neonatal outcome		
Birth weight (g)	3033.49 ± 643.81	3244.7 ± 562.75
Female sex (%)	44.12	37.04
1 min Apgar score	8.7 ± 0.61	8.75 ± 0.56
5 min Apgar score	9.81 ± 0.4	9.79 ± 0.42
Placenta weight	662 ± 128.69	695 ± 167.06

Data are presented as mean ± SD. * *p* < 0.05; ** *p* < 0.01, *t* test.
